# Microbiome of the Skin and Gut in Atopic Dermatitis (AD): Understanding the Pathophysiology and Finding Novel Management Strategies

**DOI:** 10.3390/jcm8040444

**Published:** 2019-04-02

**Authors:** Jung Eun Kim, Hei Sung Kim

**Affiliations:** 1 Department of Dermatology, St. Paul’s Hospital, The Catholic University of Korea, Seoul 06591, Korea; mdkjeun@naver.com; 2 Department of Dermatology, Incheon St. Mary’s Hospital, The Catholic University of Korea, Seoul 06591, Korea; 3 Department of Biomedicine & Health Sciences, The Catholic University of Korea, 222 Banpo-daero, Seocho-gu, Seoul 06591, Korea

**Keywords:** atopic dermatitis, microbiota, microbiome, skin, gut, therapeutic implications

## Abstract

Atopic dermatitis (AD) is a long-standing inflammatory skin disease that is highly prevalent worldwide. Multiple factors contribute to AD, with genetics as well as the environment affecting disease development. Although AD shows signs of skin barrier defect and immunological deviation, the mechanism underlying AD is not well understood, and AD treatment is often very difficult. There is substantial data that AD patients have a disturbed microbial composition and lack microbial diversity in their skin and gut compared to controls, which contributes to disease onset and atopic march. It is not clear whether microbial change in AD is an outcome of barrier defect or the cause of barrier dysfunction and inflammation. However, a cross-talk between commensals and the immune system is now noticed, and their alteration is believed to affect the maturation of innate and adaptive immunity during early life. The novel concept of modifying skin and gut microbiome by applying moisturizers that contain nonpathogenic biomass or probiotic supplementation during early years may be a preventive and therapeutic option in high risk groups, but currently lacks evidence. This review discusses the nature of the skin and gut flora in AD, possible mechanisms of skin–gut interaction, and the therapeutic implications of microbiome correction in AD.

## 1. Introduction

The human microbiome refers to the collective genetic information of microorganisms that inhabit the human body. It is considered a counterpart of the human genome, which is the collection of all genetic information in a person. With the discovery that microorganisms we carry have a huge impact on our health, the microbiome has been called the ‘second genome’ and is being widely studied [1]. The microbiome takes part in a number of human biological processes such as metabolism, epithelial development, and immunity. Long standing diseases such as obesity [2], inflammatory bowel diseases [3], diabetes mellitus [4], allergic rhinitis [5], and atopic dermatitis (AD) [6] are reportedly linked with the human microbiome in a non-causal manner.

Studies that are conducted to identify the dynamics of the bacterial population mostly use a metagenomic approach which is relatively easy and cheap. The use of whole-metagenome shotgun sequencing allows unrestricted access to genes of all flora present in a sample. As an alternative, next-generation sequencing (NGS) can be performed, which simultaneously analyzes thousands to millions of 16S ribosomal RNA gene amplicons on bacteria and archaea [7]. Increasing throughput and decreasing costs associated with DNA sequencing, along with the development of analyzing tools, made these approaches feasible to query microbial communities in health and disease states. Importantly, these methods do not rely upon cultivation of the microorganism, thus eliminating biases associated with culture-based techniques.

AD is a chronic, inflammatory skin condition with prominent itching. AD starts in early childhood and is usually the first manifestation of the atopic march, progressing to asthma, allergic rhinitis, and allergic conjunctivitis. AD has a complex pathophysiology which includes a skewed response towards Th2 immunity, and defects in the innate immune system. The emergence of FLG as a risk allele for atopic disease also shifted weight on the role of the skin barrier in AD pathogenesis [8]. The prevalence of AD is increasing [9,10,11,12]. Although AD runs in families, it is impossible to explain the increased prevalence of AD with genetics alone. Factors predisposing to AD may be smaller family size, urban settings, and Western diet, which affect both the skin and gut microbiota.

The microbiome has a well-documented role in AD. The crucial interaction between flora and humans in AD is best presented through the hygiene hypothesis [13]. This theory implicates that, in modern sanitized living conditions, there is reduced microbial exposure early in life, which results in inadequate immune priming. A child’s early microbiota has protective influence on the immune system from allergic over-sensitization. In contrast, poor development or imbalance of the microbiome is known to affect the cutaneous immune response in a way that children are predisposed to a number of immune conditions, such as AD, with frequent secondary skin infections. The interaction between the microbiome and the immune system in AD patients extends our knowledge on the pathogenesis of AD and is changing the traditional concept of antibiotic therapy. In this review, we discuss the roles of skin and gut flora in AD development, manifestation, and attenuation.

## 2. Skin Microbiota and AD

The skin is a shelter to a countless number of microbial communities which live on the tissue surface, as well as the appendages, such as the sweat glands and the hair follicle. Across the skin surface, 1 million bacteria are found per square centimeter with over 10^10^ bacterial cells in total [14]. There is topographical diversity of the bacterial populations on the skin, which depends on the micro-environment (temperature, age, amount of sebum, sweat, etc.) [15]. Sebaceous sites are full of lipophilic *Cutibacterium* (formerly *Propionibacterium*) species, while moisture-loving *Corynebacterium* and *Staphylococcus* species are present in great quantities in moist areas. The fungus *Malassezia* is abundant on the trunk and arms [16]. The human skin flora, possibly the most diverse within the body, are reckoned to be crucial in host defense. Commensal skin flora protect humans from pathogens and help maintain the delicate balance of the immune system between effective protection and damaging inflammation. Commensal flora such as *Staphylococcal epidermidis* (*S. epidermidis*) produce antimicrobial substances that fight off pathogens whereas *Cuticabacterium acnes* (*C. acnes*) uses the skin lipids to make short-chain fatty acids which dampens microbial threats [17]. *Cutibacterium* and *Corynebacterium* also reduce *Staphylococcus aureus* (*S. aureus*) by forming porphyrin [18] (findings from in vitro and animal studies) (Table 1).

### 2.1. Skin Microbiota in AD

In [19], it was reported that the diversity of healthy skin microbiota is prominently higher in the younger population than in adults (alpha diversity, *p* = 0.01) and sharply different between the two age groups as shown by beta diversity. While great quantities of *Streptococcus*, *Rothia*, *Gemella*, *Granulicatella*, and *Haemophilus* are present in young children; *Cutibacterium*, *Lactobacillus*, *Anaerococcus*, *Finegoldia*, and *Corynebacterium* were more common in adults. Significant differences in skin microbiota were also identified between AD children and adults (beta diversity, *p* < 0.001). AD patients were found to carry the 20 genera that were prevalent in the healthy population [19]. The prevalence of AD among children is 20 to 30 percent, whereas that of adults is merely 3%. Microbial shift may potentially contribute to the age-related reduction in AD by suppressing the growth of *S. aureus*. Adult-associated skin commensals *Cutibacterium* and *Corynebacterium* [20] harbor genes involved in porphyrin metabolism [21] which theoretically can reduce *S. aureus* infection [18] (findings from in vitro and animal studies). In addition, adult skin flora secrete metabolites with antimicrobial properties, which in turn block the growth of *S. aureus* as shown in in vitro and mice study [22,23].

AD patients are reported to carry *S. aureus* on their skin at rates varying from 30–100%, whereas *S. aureus* is only found in 20% of healthy people [24]. According to a meta-analysis, *S. aureus* carriage is different even within the same AD patient, ranging between 39% on non-lesional skin and 70% on lesional skin [25]. *S. aureus* density is known to correlate with disease severity, regardless of the site (both lesion and non-lesional skin) (causality not proven) [25]. In addition to *S. aureus*, other *Staphylococcus* species (*S. epidermidis* and *S. haemolyticus)* are increased on AD involved sites [26,27,28,29]. The skin flora shows low microbial diversity during an AD flare regardless of age [27]. In particular, inflamed AD skin is associated a decrease in the genera *Cutibacterium*, *Streptococcus*, *Acinetobacter*, *Corynebacterium*, and *Prevotella* and an increase of *Staphylococcus*, especially, *S. aureus* [27] (Table 2).

In one study [30], the abundance of *C. acnes* was found to correlate negatively with *S. aureus* where the growth of *S. aureus* and *S. epidermidis* was blocked by the fermentation products from *C. acnes* (confirmed by culture study). Bacterial diversity is also closely linked with the quality of the skin barrier, portrayed by transepidermal water loss (TEWL) and pH level in canine AD [31]. With the help of shotgun metagenomic sequencing, AD patients were found to carry a single strain of *S. aureus* during severe flares. Interestingly, the *S. aureus* strains extracted from AD patients are different from those of unaffected carriers [32]. Whereas most AD patients carry the clonal complex (CC1) strains, the CC30 strains are common among asymptomatic nasal carriers in the normal population [33,34,35]. The skin flora becomes more diverse following AD treatment [27].

The number of comprehensive studies on mycobiota is substantially lower than that made on bacterial microbiota. Zhang E. et al [36] reported that among the three taxonomic categories of fungus (non-*Malassezia* yeast, *Malassezia*, and filamentous fungi), *Malassezia* species are the most noticeable in AD subjects, accounting for 63–86% of the clones. *Malassezia restricta* (*M. restricta*) and *Malassezia globosa (M. globosa*) are the two major subtypes isolated within the genus *Malassezia.* The ratio of these two *Malassezia* species differs by AD severity. AD patients with mild to moderate severity show predominance of *M. restricta* over *M. globosa*, whereas the ratio approximates to 1 in severe AD patients. On whole, the non-*Malassezia* yeast (ex. *Cryptococcus liquefaciens Candida albicans*, *Cryptococcus diffluens*) in AD patients were more diverse than that found in the healthy population [37].

### 2.2. Epidermal Barrier Status Impacts the Composition of the Skin Microbiota

AD is a long-standing inflammatory skin disease typified by epidermal barrier dysfunction which can affect the bacterial community of the skin. The stratum corneum consists of dead cells (brick) and a lipid matrix (mortar), which take part in epidermal permeability. Important lipids of the stratum corneum are free fatty acids (FFA), ceramides, and cholesterol. FFAs are crucial for the skin barrier and their chain lengths are much shorter in AD skin. Integrated analysis has revealed a strong association between microbiome and lipidome composition where the abundance of *Cutibacterium* and *Corynebacterium* positively correlated with levels of long-chain unsaturated FFAs in the epidermis [38]. The levels of FFA 16:1 and FFA 18:1 were also found to be significantly lower in the AD *S. aureus* (+) population compared to AD *S. aureus* (-) patients [39]. Exogenous FFA 16:1 is reported as a potent bacterial growth inhibitor (in vitro finding) [40], which suggests that FFAs are responsible for antimicrobial defense [39]. The proportion of ceramide 1, which is a linoleate carrier that functions as the skin’s water-barrier, is low in AD patients. The level of very-long chain ceramide is also decreased by a large amount in AD *S. aureus* (+) patients when compared to the AD *S. aureus* (-) population and negatively correlates with TEWL [39].

Filaggrin is a crucial component of the skin barrier and its loss-of-function mutation is related to AD as well as asthma [41]. In patients with established AD, filaggrin deficiency, either genetic or derived from Th2 dominant conditions have been shown to cause defects in corneocytes [42]. AD *S. aureus* was found to bind strongly to these corneocytes in a clumping factor B-dependent manner in an ex vivo study [33]. Filaggrin deficiency in AD is also associated with a higher pH, a condition favorable to *S. aureus* growth (in vitro study finding) [43].

In AD patients, the activity of serine protease (specifically kallikreins) are increased [44,45]. Hyperactive kallikreins are known to alter cathelicidin and filaggrin processing and increase Protease activated receptor 2 (PAR-2) activity (in vitro and mice study) [46,47,48,49]. This in turn, compromises the skin barrier and increases *S. aureus* colonization.

**Figure 1:**
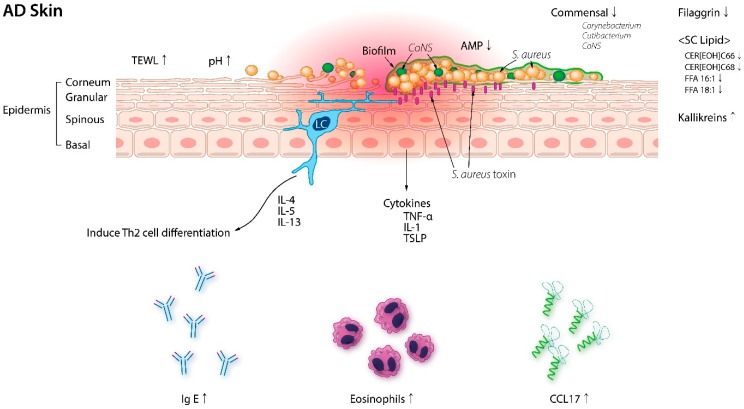
Epidermal barrier disruption in AD skin. Trans-epidermal water loss (TEWL), pH, serum IgE, serum thymus and activated cytokine (TARC/CCL17), and eosinophils are significantly elevated in AD patients. Filaggrin and stratum corneum (SC) lipid composition, and serine protease (Kallikreins) are also altered in AD, allowing *S. aureus* colonization. With the decrease in coagulase-negative *Staphylococci* (CoNS) and its antimicrobial peptides (AMP), *S. aureus* proliferates and also forms biofilms. AD: Atopic dermatitis; CER: Ceramide; FFA: Free fatty acid; IL: Interleukin; *S. aureus*: *Staphylococcus aureus*; TNF: Tumor necrosis factor; TSLP: Thymic stromal lymphopoietin.

The protective role of the skin barrier is attributable to antimicrobial peptides (AMPs) which are small peptides available in large quantities within the skin. AMPs such as β-defensins, cathelicidin, and dermicidin are less abundant in AD skin under to the presence of Th2 cytokines, which makes the skin permissive to *S. aureus* colonization [50] (Figure 1). Interestingly, coagulase-negative *staphylococci* (CoNS) strains that express AMPs are abundant in normal skin but rarely detected in AD lesions [51]. Recently, Nakatsuji et al. [51] reported that AMPs produced by commensal CoNS species *Staphylococcus epidermidis* and *Staphylococcus hominis* synergize with the human AMP cathelicidin in killing *S. aureus* (mice study) which suggest that interactions between microbial communities within the skin play a central role in the pathogenesis of AD.

Fungal microbiota is also affected by epidermal barrier integrity. Skin barrier dysfunction show positive correlation with AD severity and TEWL. It has negative correlation with skin pH. Significant alterations in skin pH, TEWL, and lipid composition in AD are thought to play a great part in change of fungal microbiota.

### 2.3. Staphylococcal Biofilms in AD

The severity of AD is significantly influenced by the ability of *S. aureus* isolates to form biofilms [52], which is a bacterial assemblage attached to the surface and enclosed in an extracellular matrix. Recent studies have reported Staphylococcal biofilms colonizing eccrine ducts adjacent to AD lesions [53], where both early (IL-1β) and late (IFN-γ) AD inflammatory cytokines induce growth of biofilm-growing *S. aureus* strains in a concentration dependent manner (in vitro study) [52]. The importance of staphylococcal biofilms in the pathogenesis of AD was highlighted by a number of in vitro studies which demonstrated significant impacts of staphylococcal biofilms on immune evasion as well as the differentiation and apoptosis of keratinocytes [54]. Neutrophils are known to be inhibited by *S. aureus* via neutrophilic lysins such as α-toxin, which is upregulated upon *S. aureus* biofilm formation [55]. In addition, macrophage phagocytosis is inhibited by specific proteins secreted from *S. aureus* biofilms (i.e., alpha toxin, Leukocidin A, Leukocidin B) [56,57]. Next to the immune evasion properties that lead to recurrent, hard-to-treat infections, staphylococcal biofilms exert direct effects on keratinocytes [58]. A potentially significant impact of *S. aureus* in AD patients is its ability to trigger apoptosis in keratinocytes with subsequent release of thymic stromal lymphopoietin (TSLP) [59]. TSLP secretion results in a strong itch response [60] and can also induce dermal dendritic cell activation and recruitment of Th2 cells that secrete IL-4 and IL-13, which have a suppressive effect on AMPs [61]. The biofilm extracts induce keratinocytes to secrete Il-6, causing decreased expression of keratin 1 and 10, as well as filaggrin [62] which renders the keratinocytes to be more susceptible to the cytotoxic effects of staphylococcal α-toxin [63]. *S. aureus* biofilms can also be a source of proteolytic enzymes (i.e., staphopains) at the skin surface, with the capacity to cleave endogenous AMPs and interfere with the epidermal inflammatory response [64].

### 2.4. S. aureus Damages the Skin Barrier and Exacerbates AD Inflammation

Next to having excellent adhesion and immune avoidance mechanisms via biofilms, *S. aureus* has a number of powerful resources that help invade and derange the skin barrier (in vitro findings) [24]. *S. aureus* secretes a pore forming α-toxin which penetrates host cell membrane [65,66]. In the epidermis, the α-toxin forms direct pores on keratinocytes, which destroys the skin barrier. *S. aureus* also produces a number of proteases, which dissolve the stratum corneum [67]. Notably, the protease activity is magnified in settings where Th2 cytokines are present, and where filaggrin is absent [67]. In addition to protease secretion, *S. aureus* directly activates keratinocyte proteases, which includes kallikreins KLK6, 13, and 14 [68]. These serine proteases degrade desmoglein-1 during normal desquamation [68,69], but dysregulation in their activity can lead to impaired barrier function [54]. Recently, a *S. aureus* cell wall product, lipoteichoic acid (LTA) was shown to cause skin barrier damage by inhibiting the expression of epidermal barrier proteins filaggrin and loricrin [70]. This highlights the various mechanism *S. aureus* offers towards barrier destruction, increasing water loss and allowing greater exposure to external antigens [71].

*S. aureus* also presents a number of molecules that contribute to the disease via pro-inflammatory mechanisms (in vitro findings) [24]. Protein A, a surface protein originally found in the cell wall of *S. aureus*, induces an inflammatory response from keratinocytes by binding to the tumor necrosis factor receptor 1 (TNFR1) [72]. Staphylococcal super-antigens such as toxic shock syndrome toxin-1 (TSST-1) and staphylococcal enterotoxin A (SEA), SEB, SEC, trigger B cell expansion, and cytokine release [72]. In a toll-like receptor (TLR)-2/TLR-6 dependent manner, proinflammatory staphylococcal lipoproteins provoke keratinocytes to express TSLP, confirming that there are multiple routes through which *S. aureus* brings about a persistent and self-perpetuating Th2 response [73]. *S. aureus* also releases phenol soluble modulins (PSMs), which drive inflammation with sector-specific effects [24]. In the epidermis, PSM α triggers keratinocytes to produce IL-36, and causes IL-36 α-driven γδ T cell-mediated inflammation, whereas in the dermis, it activates IL-1β-operated and innate lymphoid cells-propelled Th17 inflammation [74]. PSM γ (δ-toxin) fuels dermal mast cells and promotes skin inflammation [74] (Table 3).

### 2.5. Skin Dysbiosis and AD

*S. aureus* colonization in AD skin is common and can cause the course of AD to be more complex. Despite the knowledge, the causal association between dysbiosis and AD is not yet clarified. The latest murine studies show that cutaneous flora can impact the evolution of the skin immune system and disease [75,76,77]. Finding out whether cutaneous flora take part in the beginning of AD may give us a chance to prevent atopic disorders. In order to address this, large-scale prospective studies analyzing the microbiota in a longitudinal manner were performed. Kennedy et al. [78] reported that two-month-old infants who were diagnosed with AD later on carried a significantly lower number of commensal *Staphylococcus* species on the antecubital fossa compared to those who were unaffected at 12 months. They failed to detect substantial colonies of *S. aureus* on infants who later on developed AD, and made conclusions that *S. aureus* colonization follows the onset of AD. Meylan et al. [79] presented a longitudinal study of 149 infants during their first 2 years to find increased colonies of *S. aureus* before the start of AD. In the same study, *Staphylococcus hominis* was found to be significantly less copious in those who developed AD later-on [79]. Although there are conflicting data on *S. aureus*, its role in AD initiation is supported by several lines of evidence. *S. aureus* strains isolated from AD patients secrete various exotoxins and children with severe manifestations were found to carry toxigenic strains of *S. aureus* more frequently [80]. A causal relationship between *S. aureus* and AD was also found in murine experiments. Kobayashi et al. [77] showed that *S. aureus* inoculation accelerates the development of an eczema similar to AD. Early exposure to commensal *Staphylococci* may protect one from developing AD later-on. Having antigen-specific tolerance to commensal flora rely on early colonization, indicating that there is a sensitive period for generating regulatory T cells to these bacteria (mice study) [76]. In comparing infants with an older population, activation of TLRs results in less TNF-α and greater production of IL-23 and IL-6 [81]. The adaptive immune system matures alongside with regulatory T cells which are found in higher quantity during fetal life and infancy [82,83,84]. Although these characteristics put neonates at higher risk of disseminated infection, they also stimulate immune tolerance to foreign and self-antigens, thereby blocking unfavorable inflammation [24]. Scharschmidt et al. [76] applied a commensal *Staphylococcus* species on neonatal mice to successfully produce immune-modulation. It remains unknown whether early exposure to commensal *Staphylococci* in humans has a similar effect. Further research is required to fully understand its influence on AD development. The longitudinal studies have focused on *S. aureus* and there is currently little information on the role of fungi, viruses, and Gram-negative bacteria in AD development.

### 2.6. Effect of Treatment on the Skin Microbiota in AD

Microbial diversity and the proportion of *Staphylococcus* varies among specific AD disease states. Kwon et al., evaluated changes in lesional and non-lesional skin microbiota during AD treatment (narrow band ultraviolet B (NBUVB) phototherapy and topical corticosteroids versus. topical corticosteroids alone) to find a drastic increase in microbial diversity and decrease in *S. aureus* proportion on lesional skin after treatment [85]. Shannon diversity of non-lesional skin of the NBUVB + topical corticosteroids group increased at week 6 while that of the topical corticosteroids alone population remained similar. The effect of NBUVB therapy in AD is well documented in various studies [86,87], and some research findings suggest its protective role to be achieved by restoring dysbiosis of AD skin. Previous studies have found that ultraviolet phototherapy reduces *S. aureus* colonization in lesional AD skin [88] and reduces toxin production from *S. aureus* [89]. Ultraviolet exposure also induces an AMP cathelicidin production in AD skin, which guards the skin from *S. aureus* [90]. In another study with AD and healthy children, the influence of topical steroids plus dilute bleach baths and topical steroids alone were evaluated [26]. Both treatment arms normalized the bacterial composition of lesional skin to that of non-lesional skin, and the diversity of the skin flora was comparable to that of control skin. Bleach bath did not have additional impact on the skin flora. In a study by Kong et al. [27], the nature of bacterial communities during AD disease states was analyzed to look into the microbial characteristics related to AD flares (defined as worsening disease for more than 24 h regardless of any intervention before sampling) and improvement post-treatment. Diversity of the skin flora during AD flares correlated with the presence or absence of recent AD treatments, where even sporadic treatment (use of topical calcineurin inhibitors/steroids/ or antibiotics in the past 7 days and/or consumption of oral antibiotics within 4 weeks before sampling) was linked to reduction in *S. aureus* and higher bacterial diversity. Since sporadic treatment flares present as clinically worsened disease, the authors suggest that continuous treatment over an ample period of time is necessary to reach the ‘resolving flare’ state, where there is full recovery on microbial diversity and reduction in *Staphylococcus* population, typical of a true post-flare.

## 3. Gut Microbiota and AD

The bacterial cells within the human gut overpower the host’s cell in number by a factor of 10 and the genes encoded by these bacteria outnumber their host’s genes by more than 100 times [91]. The microbes linked with the human digestive tract are cited as the gut microbiota. Extensive research has been made on the human gut microbiota and its role in disease and healthy state, identifying the interaction of gut microbiota with nutrition, metabolism, physiology, and immune function.

Microbes colonize the neonatal gut starting at birth and continues to evolve in species abundance until the infant is 2–3 years old, at which point the flora becomes adult-like [92]. A number of studies show that the mode of delivery influences gut microbiota development in early life [93]. Vaginal delivery causes exposure to maternal vaginal flora, made up of commensal organisms often found in the lower GI tract [94]. Certain *Bifidobacterium* and *Bacteroides* species with health promoting effects are highly abundant in these infants, allowing downregulation of inflammatory responses [95,96,97]. On the other hand, an aberrant microbial community, predominant with *Streptococcus* species, *Staphylococcus* species [98], and *C. difficile* [99] is found in infants delivered by caesarean section. Infant feeding methods, such as formula feeding and breast milk feeding strongly influence the composition of gut microbiota in early life. Bottle-fed infants are enriched with *Escherichia coli (E. coli)* and *Clostridium* (ex. *C. difficile*) [100] whereas breast milk-fed infants show abundance of a specific type of *Bifidobacterium* [101]. The introduction of solid foods produces a dynamic shift in the gut flora from *Bifidobacterium*-dominant to *Bacteroides* and *Clostridium* dominant composition [102,103,104]. These microbiota persist throughout adulthood in the absence of disturbances such as serious illness, change in diet, or prolonged use of antibiotics [105].

**Figure 2:**
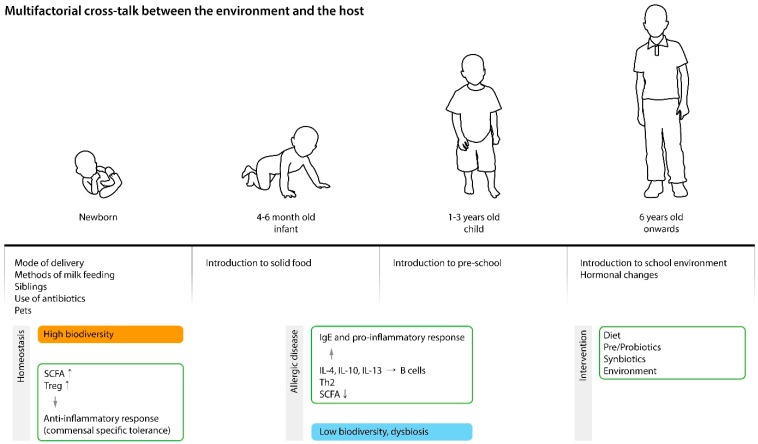
The complex interaction between the environment and the host. High microbial diversity associated with vaginal delivery, breast feeding, interaction with siblings and pets increases regulatory T cells (Treg), short chain fatty acids (SCFAs), and immune tolerance. Low biodiversity early in life affects the host immune system which is likely to cause a proinflammatory response. Intervention with pre/pro- and synbiotics and vitamin D may favorably influence the intestinal environment. IgE: Immunoglobulin E; IL: Interleukin; Th2: T helper 2 cells.

Intestinal bacterial colonization in early life (first 3 years) has great impact on the host immune system which affects host health and disease later in life. Proper immune system development is highly dependent on intestinal bacteria as shown by the immune function loss in germ-free mice [106,107,108]. Prior human and animal studies have shown that gut flora and their metabolites (i.e., short chain fatty acids (SCFAs)), take active part in both B cell and T cell proliferation and differentiation, thus inducing protective antibody responses [109] (Figure 2).

### 3.1. Gut Microbiota in AD

The nature of intestinal microbiota in AD patients and their sex- and age-matched controls have been previously analyzed. In the study by Watanabe et al. [110], the *Bifidobacterium* counts in AD patients were significantly lower (7.6 ± 5.0 years) than in healthy individuals. Furthermore, *Bifidobacterium* count and percentage differed by the disease state, where lower numbers were found in those with severe AD but not in patients with mild atopic symptoms. In direct contrast, *Staphylococcus* was more abundant in AD patients than in healthy individuals. Song et al. [111] showed that enrichment of *Faecalibacteriuim prausnitzii* (*F. prausnitzii*) subspecies (F06) is highly related to AD (causality not proven). Fieten et al. [112] identified a microbial signature which distinguishes AD children with food allergy. The fecal microbiota of AD children with food allergy had relatively more *Bifiobacterium pseudocatenulatum (B. pseudocatenulatum*), and *E. coli* and less *Bifiobacterium adolescentis* (*B. adolescentis*)*, Bifiobacterium breve* (*B. breve*), *F. prausnitzii*, and *Akkermansia muciniphila* (*A. muciniphila*) than of those with no food allergy. The microbial diversity (according to the Shannon index) was largely indifferent between AD children with/without food allergy.

### 3.2. Association between Gut Dysbiosis and AD

Bacterial colonization of the intestines and establishment of gut flora in infancy are closely linked with immune system development [113]. As a matter of fact, the findings from numerous cohort studies suggest that aberrant gut microbiota precede the onset of atopic disease [114]. AD, the skin manifestation of atopy, is usually the first step in the atopic march. Some cohort studies found that AD infants have a lack of bacterial diversity in addition to low quantities of *Bifidobacterium* and *Bacteroides* and high levels of *Enterobacteriaceae* [115,116,117,118,119]. Fujimura et al. [120] claimed that infants with higher risk of atophy such as AD and asthma show low levels of *Akkermansia*, *Bifidobacterium*, and *Faecalibacterium*, and high quantities of *Candida* and *Rhodotorula.* Atopic infants (those who are skin prick test positive at 12 months) reportedly had elevated levels of *Clostridium* and reduced levels of *Bifidobacterium* in their stools at 3 weeks of age [121]. *C. difficile* colonization in early life also led to subsequent AD development in other studies [100]. Ismail and colleagues [122] showed that, among those with high risk of allergic disease, infants who had diverse gut microbiota at the age of 1 week had a lower risk of AD. Reduced biodiversity of the gut flora and delayed *Bacteroidetes* colonization in one-month-old infants also have been associated with subsequent AD [115]. Similar to skin commensals, differences in gut microbiota at species level are thought to be related to the disease. Infants with allergy more often carry *B. adolescentis* [123,124,125], while in healthy infants, *B. bifidum* is the dominant strain among the *Bifiobacterium* population [123,124]. In a prospective large-scale birth cohort study (KOALA) [100], AD infants were more heavily colonized with *C. difficile* and *E. coli* compared to those without AD.

As mentioned earlier on, the gut flora established during infancy, which includes the weaning period, is critical for immune system development [126]. Transformation of naïve T cells into different types of Th cells such as Th1, Th2, and Th17 or Forkhead box P3 (Foxp3)+ Treg cells is largely dependent on the gut flora [127,128]. Treg cells prevent naïve T cells from differentiating into Th cells [129,130] and control inflammation by downregulating cellular activities of mast cells, eosinophils, and basophils. It also suppresses IgE production and induces IgG4 [131]. *Bifidobacterium*, *Lactobacillus*, *Clostridium*, *Bacteroides*, and *Streptococcus* [132], and their metabolic products butyric acid and propionic acid, are well-known for their ability to induce T reg cells [133]. Regulatory T cells develop in the thymus (tTreg) but naïve T cells may also transform into Treg cells in the periphery (pTreg). From the two, extrathymically generated regulatory T cells (pTreg) control mucosal Th2 inflammation [134]. Recent studies report that the gut flora and *Bacteroides fragilis* symbiosis factor (polysaccharide A) promote pTreg cell generation [134,135]. As for the T helper cells, each type of Th cell plays its own part in shaping-up the immune response and generates cytokines to block the activity of other Th cells. Th17 cells secrete IL-17, IL-17F, and IL-22 and take part in maintaining the barrier function of the GI tract and contribute to pathogen clearance at the mucosal surface. Unmethylated DNA [136], bacterial flagella [105], and adenosine triphosphate (ATP) [108] are key mediators that drive Th17 differentiation. The mutual interaction between Th1 and Th2 cells is important for homeostasis and either Th1 or Th2 skewing can lead to chronic inflammation and autoimmune or allergic conditions [105].

**Figure 3:**
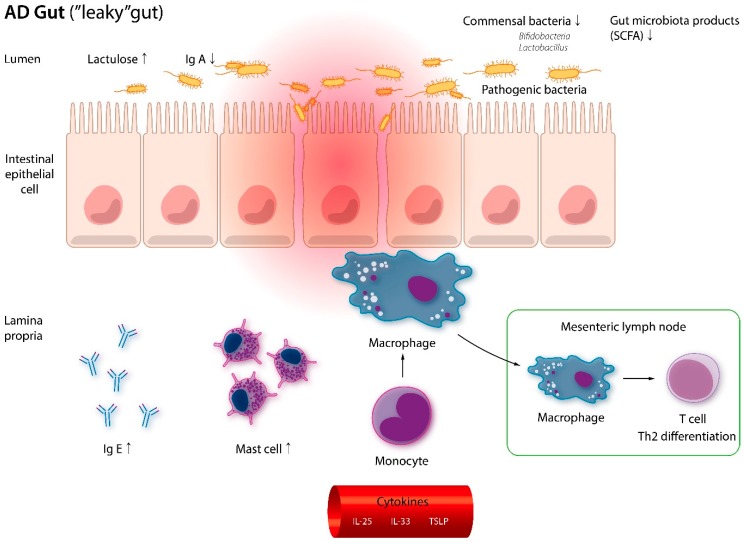
Muscosal barrier disruption in AD. Patients with AD have dysbiosis and less short-chain fatty acids (SCFAs) in the gut. In response to pro-inflammatory cytokines, monocytes migrate and differentiate into macrophages. Greater access to luminal antigen also causes T cells to transform into Th2 cells in the draining lymph nodes. Immunoglobuin E (Ig E) and mast cells are also more abundant in the lamina propria. IgE: Immunoglobulin E; IL: Interleukin; Th2: T helper 2 cells; TSLP: Thymic stromal lymphopoietin; IgA: Immunoglobulin A.

In two Korean metagenomic studies [111,137], significant dysbiosis of *F. prausnitzii* species was found in the fecal samples of AD patients. Concurrent decrease of SCFA, which takes part in keeping the integrity of the epithelial barrier and poses anti-inflammatory effect, was also noted. The ‘leaky gut’ in AD patients propels skin inflammation by enabling the penetration of toxins, poorly digested food, and microbes, into the systemic circulation. As they reach the skin, a strong Th2 responses is initiated, causing significant tissue damage [106,138,139] (Figure 3).

The gut microbiota shape-up the skin flora as well. SCFAs (i.e., propionate, acetate, butyrate) are end products of dietary fiber fermentation in the gut and are known to take an important part in determining the microbial composition of the skin which is closely linked with the cutaneous immune defense mechanisms [140]. *Cutibacterium*, produces acetate and propionic acid in the gut, which are SCFAs. Propionic acid and its esterified derivatives suppress the growth of methicillin-resistant *Staphylococcus aureus* USA300 (in vitro study) [22,141,142]. In the meanwhile, cutaneous commensals such as *S. epidermidis* and *C. acnes* tolerate wider SCFA shifts than others. Together, the findings suggest that there is a mutual interaction between the gut and skin [140].

In a German study with high-risk neonates to AD [143], the mode of delivery had profound effects on the incidence of AD, which is likely due to intestinal flora perturbation (with an abundance of Clostridium cluster I). The same study also claimed that there is beneficial influence from older siblings on gut microbiota development, which subsequently leads to less AD. The mediation analyses results suggest that through microbial modulation, factors such as the presence or absence of siblings, and the mode of delivery and can influence the risk of AD.

## 4. Impact of Probiotics/Prebiotics on AD

Since AD is associated with dysbiosis, selective modulation of the host flora to treat AD has become a center of interest. The gut flora may be normalized by using pro-, pre-, or synbiotics. Probiotics are live bacteria and yeasts recognized as having various health benefits. When ingested, probiotics provide benefits by interacting with the intestinal flora, and also, when put on the skin by topical means, work by modulating the skin microbiota [144]. Prebiotics contain nonliving indigestible fibers that encourage growth and activity of beneficial microorganisms [145]. Synbiotics are a synergistic combination of probiotics and prebiotics which promote healthy skin and a balanced set of gastrointestinal bacteria [146].

**Figure 4:**
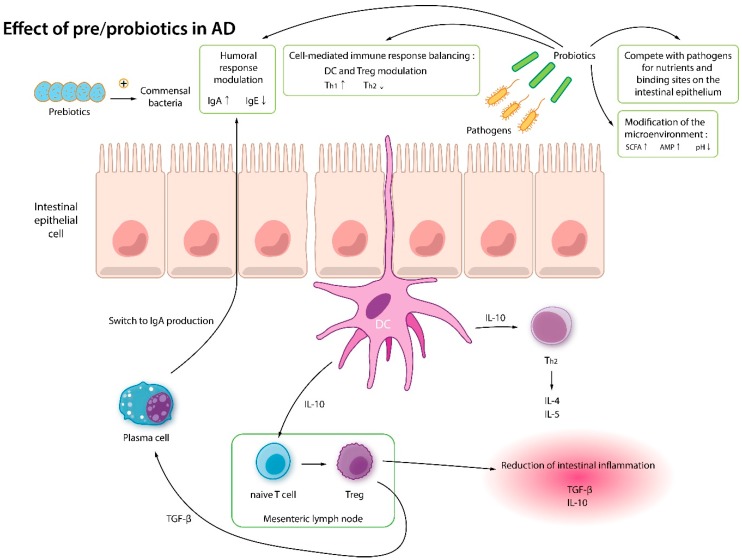
Immune mechanisms of pre- and probiotics. Prebiotics feed the commensal bacteria and protiotics. Probiotics modulate the humoral response (increase IgA and decrease IgE), balance cell-mediated immune response (increase Treg cells and decrease Th2 response), compete with pathogens, and modify the microenvironment. SCFA: short chain fatty acid; AMP: anti-microbial peptide; DC: Dendritic cell; IL: Interleukin; TGF: Tumor growth factor; IgE: Immunoglobulin E; IgA: Immunoglobulin A; Th1: T helper 1 cells; Th2: T helper 2 cells.

Gram-positive *Bifidobacterium* and *Lactobacillus* are popular probiotic families. They lack lipopolysaccharides which cause inflammation and release active molecules that help maintain a healthy gut and skin. Probiotics modulate the immune system by encouraging regulatory T cell differentiation, and also by producing anti-inflammatory cytokines (TGF-β and IL-10) [147,148]. In a mice study, intraperitoneal injection of a *Lactobacillus* strain caused an increase in IL-12 and decrease in IgE, which in theory, can be beneficial in anaphylaxis, food allergy, and AD [149]. *Lactobacillus* has been shown to accelerate skin barrier recovery and inhibits skin inflammation related to substance P [150]. *Bifidobacterium* exerts antipruritic effects by producing kynurenic acid, which is a metabolite that possesses neuroactive activity (i.e., antipruritic, antinociceptive) [151]. A recent probiotics trial with human-origin *Lactobacillus* and *Enterococcus* revealed that probiotics can increase the production of SCFA, thereby strengthening the mucosal barrier [140,152] (Figure 4).

Unfortunately, the effect of probiotics in human studies show contradictory results. According to a double-blind placebo-controlled trial, *Lactobacillus rhamnosus* GG (*L. rhamnosus* GG) taken at pregnancy prevented half of the high-risk infants from developing AD at 2 years, and this effect was kept constant at the age of 4 years [153]. Another group reported that weaning infants who were given *Bifidobacterium lactis* (*B. lactis*) Bb-12 or L. strain GG developed AD which was clinically less severe [154]. However, in a more recent study, supplementation of *L. rhamnosus* GG during pregnancy and early infancy did not reduce the incidence and severity of AD in affected children. Moreover, probiotic supplementation increased the episodes of wheezing bronchitis. A recent Cochrane Review assessed the effects of probiotics for treating patients of all ages with eczema and questions the beneficial effects of probiotics on AD [155]. According to the analysis, probiotics made little or no difference in participant- or parent-rated symptoms of eczema. There was also no evidence that probiotics make a difference in quality of life (QoL) for patients with eczema. Probiotics slightly reduced the investigator-rated eczema severity scores, but this again was not clinically-sufficient. Therefore, use of probiotics for the treatment of eczema is currently not evidence based. While the meta-analysis does not support the use of probiotics for AD, we would like to point out that variations between the species and strains employed as treatment may have contributed to the overall variance in response.

Certain moisturizers containing biomass of non-pathogenic bacteria *Vitreoscilla filiformis* was beneficial in normalizing the skin microbiota in AD and significantly reduced the episodes of flare-up [156]. Topical application of CoNS to AD skin (humans and mice) reduced skin colonization by *S. aureus* [51,157]. Topical application of a Gram-negative organism *Roseomonas mucosa* improved AD by decreasing itch, clinical severity, and the use of topical steroids [158]. The mechanism of action of these topically applied environmental bacteria are not yet known although they clearly exert an anti-inflammatory effect. [159]. So far, only a handful of studies have been conducted with topical probiotics. Further studies reporting long-term data on eczema symptoms and QoL should be performed to determine its true effects.

## 5. Vitamin D, Microbiota, and AD

**Figure 5:**
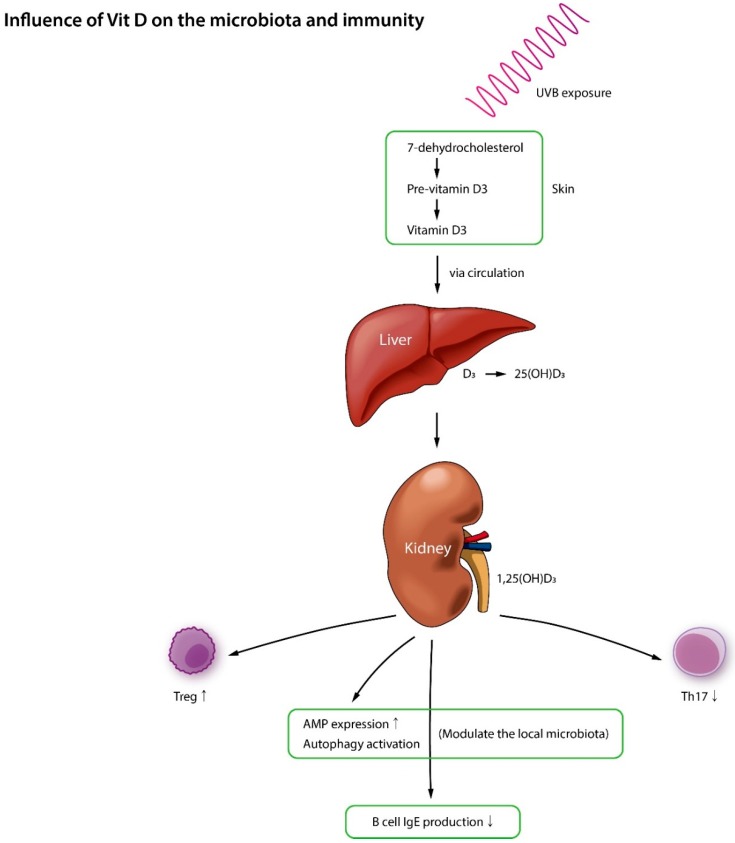
Vitamin D controls autophagy and the production of antimicrobial peptides which can help normalize the microbiota. It also regulates innate and adaptive immunity in various ways.

Recent research suggests that vitamin D blood levels inversely correlate with AD severity and that vitamin D significantly reduces the severity of AD [160,161]. However, due to the differences in clinical parameters and study design, the topic is still controversial, and researchers do not have clear evidence to recommend vitamin D supplementation to AD patients yet. In terms of the proposed mechanism of action, vitamin D controls autophagy as well as the production of AMPs (e.g., β-defensin and cathelicidin) which results in the inhibition of *S. aureus* and a change in microbial community [162]. The epidermal barrier and the immune system are known to be closely intertwined, and with its ability to accelerate epidermal barrier recovery, vitamin D can potentially stabilize the immunological integrity of gut and skin [163]. Vitamin D also regulates innate and adaptive immunity in a number of ways such as by encouraging mast cells to produce an anti-inflammatory cytokine IL-10, blocking the release of Th1 pro-inflammatory cytokines, preventing monocytes from expressing Toll-like receptors, reducing dendritic cell activity with lipopolysaccharides, and by cutting down the release of IgE, all of which hints that vitamin D is at least partially involved in AD and other inflammatory skin conditions [164] (Figure 5).

In one study [165], maternal vitamin D supplementation was found to help colonize the important bacterial taxa within the infant’s gut. Since gut flora homeostasis influences AD development, the possibility of vitamin D as a novel supplement to pregnant women and infants should be further studied.

## 6. Air Pollution, Gut Microbiota, and AD

Air pollution is the presence of harmful substances in the air caused by both natural conditions (i.e., wind, dust) and human activities (i.e., traffic, cooking, smoking) [166]. Particulate matter (PM) is a key component in air pollution and has been associated with adverse health conditions [167].

Recent evidence suggests that air pollution may be a risk factor for AD development with its ability to induce oxidative stress [168]. Lee et al. [169] reported that flexural AD was associated with air pollutants in school-aged Taiwanese children. Another study with 9–11-year-old children [170] identified that eczema was related to the concentration of major air pollutants (i.e., Carbon monoxide, Nitric oxide, PM) (causality not proven in both studies).

Mutlu et al. [171] showed that PM exposure increases the mitochondrial production of reactive oxygen species (ROS) and the release of proinflammatory cytokines, thus increasing gut permeability. This can potentially affect the dynamics of the gut microbiota resulting in dysbiosis. The proportion of *Firmicutes* were significantly low in PM exposed mice, accounting for the disappearance of *Lactobacillus* which is known to be a beneficial commensal in the gut [171]. In addition, gut exposure to PM resulted in a decrease in butyrate concentration [166] which is associated with barrier dysfunction and mucosal inflammation [172].

As mentioned earlier on in this paper, the gut microbiota develops in close interaction with the immune system [167]. An aberrant microbial colonization in early life can produce an imbalance and dysregulation of the immune system, generating a variety of pathological outcomes (i.e., AD).

## 7. Conclusions

Recent technological advances in next generation sequencing have strengthened our capability to find and categorize microbial communities of our skin and gut. Our understanding on healthy flora, and how it is shifted in the disease state (i.e., AD) is increasing steadily. However, further research would be necessary to incorporate this knowledge to therapy. Modulation of the microbiota though pre-, pro-, and symbiotic supplementation is a novel approach for improving the host’s health. Although there is currently little evidence that probiotics prevent/treat AD, it may become an alternative in the future ‘post-antibiotic’ era. Normal vaginal delivery, breast milk feeding, supplementation of vitamin D to pregnant women and infants, and restriction of antibiotics in early life may reduce the risk of AD. The microbiome is highly personal and is an important facet in human health. Precision and personalized medicine with microbiome may be applied for AD treatment in future.

## Figures and Tables

**Table 1:** Commensal flora of the major human organs [15].

	Gut	Skin
Density	10^12^/g of intestinal matter	10^6^/cm^2^
Diversity	Bacteria dominant - 7–8 Phyla of bacteria (~100 species/individual) Fungi and virus rare	Bacteria dominant - 7–8 Phyla of bacteria (~40 species/individual) Up to 10% fungi and 40% viral/phage colonization
Niche	Mucus Epithelial surfaces Crypts	Stratum corneum (surface) Appendages (e.g., Hair follicles, sebaceous glands)
Community establishment	Early life	Early life Puberty
Nutrients	Rich	Poor
Effect on the immune system	Control the development of gut-associated lymphoid structures Innate immunity activation Control the induction, function, homeostasis of the regulatory immune network Colonization resistance	Control of innate immunity - Produce AMPs (e.g., Cathelicidin, β-defensin) - Increase expression of the complement system and IL-1 Control of adaptive immunity - Increase IL-17A and IFN-γ production by dermal T cells - Control the regulatory immune network Colonization resistance - Bacteriocin, serine protease Esp, and phenol-soluble modulin (PSMs) production by *S. epidermidis* - Short-chain fatty acid and porphyrin production by *Cutibacterium*
Range of effect	Local Systemic	Local Systemic (possibly)

AMP: Antimicrobial pepetide; IFN: Interferon; IL: Interleukin, *S. epidermidis*: *Staphylococcal epidermidis*.

**Table 2:** Changes in microbial diversity in non-affected vs. lesional AD skin.

AD Skin (Non-Affected Areas)	AD Skin (Lesions)
*Actinobacteria* (phylum) *Corynebacterium* (genus) *Cutibacterium* (genus) *Rothia* (genus) *Actinomyces* (genus)	Decreased relative abundance
*Bacteroides* (phylum) *Prvotella* (genus)	Decreased relative abundance
*Proteobacteria* (phylum) *Acinetobacter* (genus)	Decreased relative abundance
*Firmicutes* (phylum) *Streptococcus* (genus) *Staphylococcus* (genus) *Granulicatella* (genus)	Decreased relative abundance of *Streptococcus/Granulicatella* Increased absolute and relative abundance of *Staphylococcus*

**Table 3:** Mechanisms of *S. aureus*-mediated AD severity.

Virulence Factors	Mechanisms for Increased AD Severity
α-toxin	Directly forms pores in keratinocytes, eroding the integrity of the epidermal barrier
Protease	Facilitate dissolution of the stratum corneum
*Staphylococcal* superantigens (SEA, SEB, SEC, TSST-1)	Trigger B cell expansion and cytokine release from keratinocytes Non-specific APC-mediated T cell activation
Protein A	Triggers inflammatory response from keratinocytes through the tumor necrosis factor receptor 1 (TNFR1)
PSM α	Stimulates keratinocyte production of IL-36, and Th17 inflammation
PSM γ (δ-toxin)	Stimulates dermal mast cells and induces skin inflammation

SEA, SEB, SEC: *Staphylococcal* enterotoxin A, B, C; TSST-1: Toxic shock syndrome toxin-1; PSM: Phenol soluble modulin; APC: Antigen presenting cells.
